# Comparison of rapid methods PBP2′ detection, ORSAB and *mecA* for detection of methicillin- resistant *Staphylococcus aureus* (MRSA) in a tertiary care centre, Chennai, India

**DOI:** 10.1186/1471-2334-14-S3-P31

**Published:** 2014-05-27

**Authors:** Latha Jayaraman, Umamaheswari Subburaya

**Affiliations:** 1 Department of Microbiology, Sri Ramachandra University, Chennai, India; 2 Department of Pharmacology, Sri Ramachandra University, Chennai, India

## Background

Early detection of MRSA in clinical specimens is imperative to prevent cross transmission, morbidity, mortality and overall cost of treatment. Additionally, rapid detection of MRSA helps in preventing superficial skin infection to become deep seated.

## Method

Fifty isolates of MRSA from different specimens like blood, urine, exudates and respiratory were collected from 2010 to 2011. MRSA screen slide latex agglutination test kit (Denka Seikan, Japan) was used for PBP2′ detection and Oxacillin Resistant Screen Agar Base (ORSAB) with supplement (Oxoid Ltd., UK) was used for detection of MRSA from the isolates with ATCC controls. The detection of *mecA* gene in the MRSA isolates was considered as the reference method for determining the sensitivity of each phenotype rapid method studied.

## Results

All the 50 isolates of MRSA were positive for *mecA* by PCR. MRSA screen for PBP2′ detection and ORSAB had a sensitivity of 93.75% and 95.8%, respectively. Chi square statistical analysis was carried out for the comparison of the rapid methods. Chi square value was found to be 3.0928 (*p* value=0.07864) and 2.0408 (*p* value =0.153127) at 5% level of significance. OR (Odds ratio) =7.4421 (95%CI=0.3744–147.9331) and OR=5.2062 (95%CI=0.2436–111.2444), respectively.

## Conclusion

In the present study, we found that the difference in sensitivity between PCR for *mecA* and the phenotype rapid methods is statistically not significant. Therefore, we conclude that phenotype rapid methods can be used for the detection of MRSA from clinical isolates in low resource health care settings.

